# Data management in balance – a decade of balancing pragmatism, sustainability and innovation at plant research center IPK Gatersleben

**DOI:** 10.1515/jib-2025-0012

**Published:** 2025-05-30

**Authors:** Danuta Schüler, Matthias Lange, Thomas Altmann, Maria Cuacos, Daniel Arend, John Charles D’Auria, Anne Fiebig, Jochen Kumlehn, Kerstin Neumann, Michael Melzer, Elena Rey-Mazón, Hardy Rolletschek, Uwe Scholz, Evelin Willner, Jochen C. Reif

**Affiliations:** Leibniz Institute of Plant Genetics and Crop Plant Research (IPK), D-06466 Gatersleben, Germany

**Keywords:** research data management, requirement engineering, plant science, data stewardship, LIMS, agile data flows and processes

## Abstract

The Leibniz Institute of Plant Genetics and Crop Plant Research (IPK) Gatersleben is a leading international plant science institute specializing in biodiversity and crop plant performance research. Over the last decade, all phases of the research data lifecycle were implemented as a continuous process in conjunction with information technology, standardization, and sustainable research data management (RDM) processes. Under the leadership of a team of data stewards, a research data infrastructure, process landscape, capacity building, and governance structures were successfully established. As a result, a generic research data infrastructure was created to serve the principles of good scientific practice, archiving research data in an accessible and sustainable manner, even before the FAIR criteria were formulated. In this paper, we discuss success stories as well as pitfalls and summarize the experiences from 15 years of operating a central RDM infrastructure. We present measures for agile requirements engineering, technical and organizational implementation, governance, training, and roll-out. We show the benefits of a participatory approach across all departments, personnel roles, and researcher profiles through pilot working groups and data management champions. As a result, an ambidextrous approach to data management was implemented, referring to the ability to efficiently combine operational needs, support daily tasks in compliance with the FAIR criteria, while remaining open to adopting technical innovations in an agile manner.

## 1 Introduction

**Figure 1:**
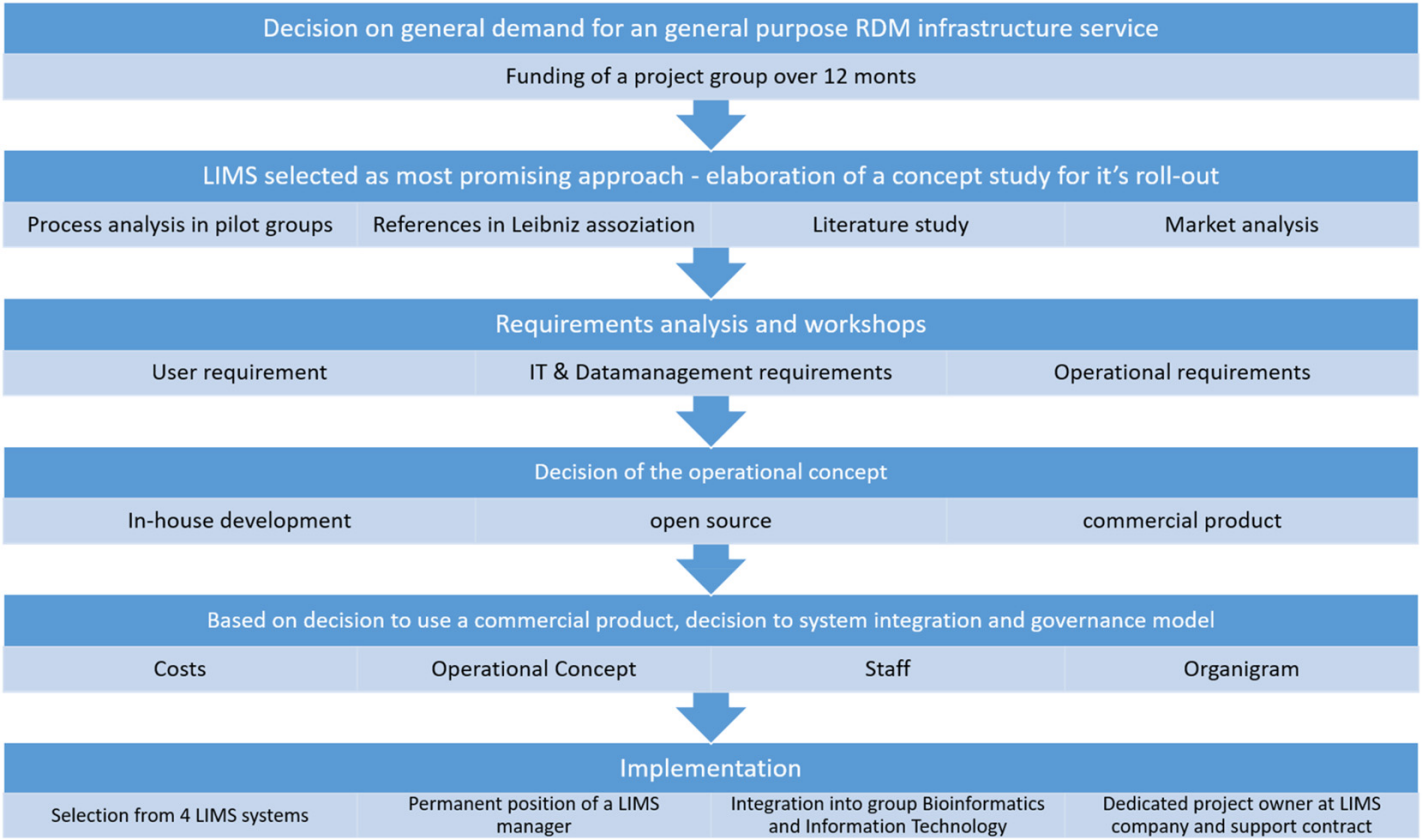
IPK roadmap to establish a research data management infrastructure.

The Leibniz Institute of Plant Genetics and Crop Plant Research (IPK) is a leading international plant science institute with a research focus on biodiversity and crop performance. Effective research data management (RDM) with the aim of creating jointly usable data spaces around the IPK genebank for plant genetic resources is an important basis for current and future innovations in basic research, applied plant breeding or for the conservation of biodiversity. Over the past decade, the IPK has initiated its digital transformation process. In subsequent years, all phases of the research data life cycle [1] and the associated FAIR principles [2] have been put into practice as a continuous process in tandem with information technology, standardisation and sustainable research data management (RDM) processes. The cross-institute RDM roadmap, as illustrated in Figure 1, started in 2008 with a project team of four cross-departmental research groups and headed by the Bioinformatics Unit of the IPK. Commissioned by the board of directors they were in charge of formulating a concept and roadmap for the strategic development of institutional RDM.

In this paper we embark on a journey to establish an institutional RDM. We present measures for agile requirements engineering, technical and organisational implementation, its governance, training and roll-out. We discuss success stories as well as pitfalls and summarise the experiences from 15 years of operating a central RDM infrastructure.

## 2 Concept study for a general purpose information management system

A project team was assembled in 2009, budgeted over one year and mandated to conduct a study to provide an objective basis for the decision-making process. This study comprised, an assessment of existing practice of data handling within the institute and a requirement assessment was conducted along two focus points: technical and operational requirements. The technical requirements included expandable data structure for mapping standard laboratory processes, intuitive, configurable user interface, multilingual capability, support for structured and non-structured data, connection of mobile devices, auditing, controlled vocabularies, search, data import and data export interfaces and data protection. Non-functional requirements were system integration, expandability, integrability in the organisational structure, roll-out model, availability and compliance with data security regulations.

The project team, which was in charge to elaborate the study, was under the umbrella of the Bioinformatics and Information Technology research group, and comprised as head a senior scientist with background as information technology engineer and two doctoral bioinformatics students, who were funded for one year, representatives of four scientific working groups, known as pilot groups, and the IPK’s Bioinformatics Coordinator. The pilot groups were selected to represent the four departments of the IPK, to ensure a high level of involvement in scientific data management practices, e.g. by means of existing software systems, lived data management processes. In addition, when putting together the study team, special care was taken to ensure that the requirements of the individual departments were covered as comprehensively as possible, while at the same time complying with the performance, sustainability and functionality demands of the information management system to be introduced. The study1As the study contains some sensitive information, it has not been published in full. An excerpt can be obtained on request. was handed over in 2010. It compiled recommendations and assessments on nine focus themes [1], 3]. An excerpt is given below.

### 2.1 Personal and organisational measures

A key recommendation of the study was that the need to pool and retain knowledge in order to secure the long-term investment in a LIMS system should be reflected in the creation of a sustainable role structure. This should be done (I) by creating dedicated job profiles of LIMS employees and (II) by recruiting and managing within a service subgroup within an established working group.

Furthermore, the roll-out was also to be combined with the design of a training programme. In the early days of training, the wide range of users and training requirements became apparent, which had to be adapted to the different needs and levels of knowledge of the respective work groups and employees. Dedicated training focal points had to be set for the following groups in particular: PhD students, scientists and technical staff. A further dimension was the specialised domain backgrounds represented at the IPK in plant biology, natural sciences and information technology.

For the introduction, customisation, configuration, system integration and operation of an IPK-LIMS, it was recommended that the following roles and work priorities be covered either by staff to be recruited or by synergies with already existing staff:

**Consulting and training** – continuous requirement analysis; collection of data management processes, 1st level support.
**Software engineering** – extensions, export and import interfaces, development tailored frontends, 2nd level support.
**Administration** – monitoring, issue management, software updates, configuration, user management, server management.
**Management** – central contact point LIMS and data management issues; update and develop research data management concepts, outreach scientific to projects, resource responsibilities.

### 2.2 Costs and expense estimation

The study highlighted the strategic effect that the introduction of LIMS as a central service is likely to impact on the structure of the research data infrastructure. The following framework points were therefore set for a resource estimate for the system roll-out:

1. incremental introduction in pilot groups (up to two years)
2. integration with IPK information systems and databases (one year)
3. allocation of long-term resources in the IPK budget and their bundling in the IPK organisational chart (permanent)
4. continuous development and maintenance (permanent)
5. integration into the institute’s training programme (subsequent to the introduction in the pilot groups)

In addition to functional criteria, aspects relating to personnel and organisational measures, the duration of an introduction and the maintenance costs incurred in the long term were included in the review. In this context, commercial systems, open source systems and proprietary in-house developments were compared. The estimated workload and expenses include investments in personnel and the number of positions required for the roles listed under 2.6. as well as the investment required in software, maintenance and operation (Table 1 – costs and expense estimation).

**Table 1:** The required RDM roles, the required number of personnel positions, estimated qualitative resource effort for a LIMS roll-out and operation.

		Commercial		Open source		In-house	
		Rollout	Operation	Rollout	Operation	Rollout	Operation
**Personnel**	Data steward	2	1	2	1	1	1
	Software engineer	1	1	2	1	3	2
	IT administrator	0.5	0.25	1	0.5	0.5	0.25
	Senior scientist	1	1	1	1	1	1
**Investment requirements**		High	High	Low	Middle	Low	Low
**Operating availability**		Low	High	Low	Middle	None	Middle
**Operating expenses**	Software engineering	High	Low	High	High	High	High
	Support	Middle	Middle	Low	High	Low	High

The study was evaluated by the board of directors and led to the decision to introduce a RDM infrastructure from a commercial provider. The chosen software vendor is a specialist in LIMS system engineering (https://www.limsophy.com/en), whose product portfolio includes an integrated “Research and Laboratory Information Management System” (RALIMS) that meets all the requirements formulated and has a high market presence in both public research institutions and private companies.

The key aspects in favour of a commercial vendor were the requirement for long-term sustainable operation, investment savings, and the total cost of ownership. Especially in light of Open Source versus Closed Source debate [4], there were primarily strong arguments to ensure compensations for personnel fluctuations in terms of knowledge drain, long-term support for software and system updates, continuous updating of interfaces to ensure technical compatibility with data collection processes. The latter includes the technical development of instruments, sensors, plant phenotyping and genotyping facilities, and continuously updated system documentation and training materials.

Furthermore, the support contract comprises a permanently dedicated project manager and software engineer on the vendor-side. This supports knowledge dissemination, reduces knowledge loss during staff turnover and strengthens the institutional LIMS operation team to scale out in case of increased staffing needs, e.g. vacation, system and scientific instrument upgrades, data flow support for research projects etc. This increased agility was, as shown in Table 1, complemented by predictable financial planning and was even more cost effective than long-term financing of in-house staff which high potential of fluctuation. This experience was made during the establishment of the IPK bioinformatics infrastructure, the genebank information system and IT services between 2002 and 2008 as a result of a federal and state funding programme. Here a central combined Bioinformatics and IT infrastructures were set up at company level. The corresponding maintenance, support and consulting contracts in place and are one pillar of continuous and stable service operation.

### 2.3 Technology and systemintegration

At the technical level, four characteristics of the RDM infrastructure were considered. First, universality, to manage experimental data and metadata, projects, instruments, and laboratory notebooks. Second, interoperability with existing in-house IT infrastructure, e.g. ORACLE database system, Microsoft Windows desktop software and compatible file store. Third, capabilities for an agnostic support of data flows and support for open format compatible bulk data imports. And fourth, the model for long-term sustainable service.

**Figure 2:**
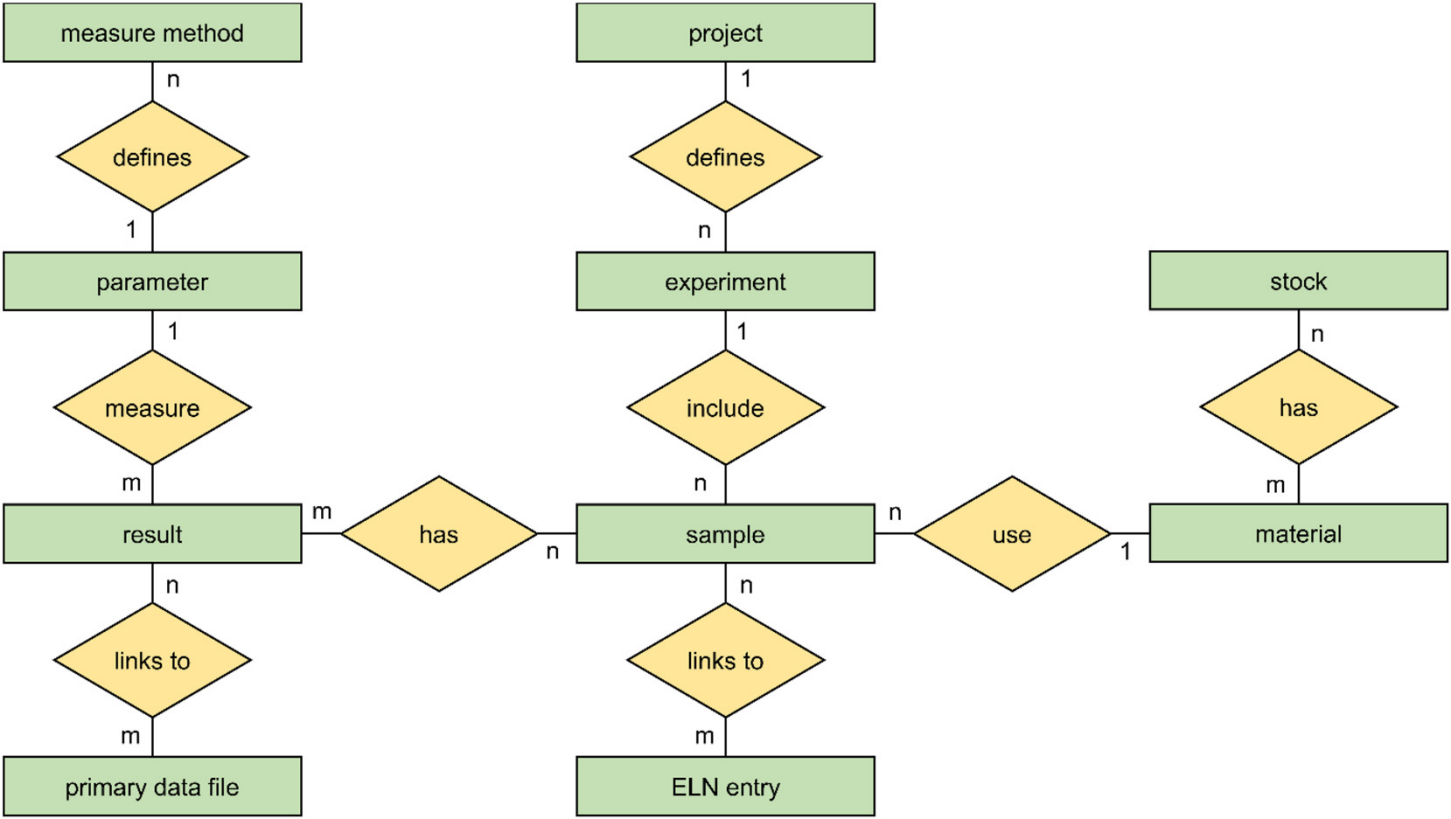
Core entities and relations of the RALIMS database structure.

The focus was on the system integrating of a RALIMS into IPK’s IT ecosystem that comprises (a) an ORACLE relational database, (b) a hierarchical storage management (HSM) system for archiving LIMS-referenced primary data files and (c) a Microsoft Windows Server Cluster for hosting the RALIMS front-end as a desktop client agnostic remote desktop application. The underlying data structure of RALIMS is generic and similar to the Investigation-Study-Assay (ISA) concept [5]. As illustrated in Figure 2, this consists of data entities and attributes that model a large part of the data generated in a research institute and are implemented efficiently as tables in an RDBMS. More details to the data structure was published in [5].

**Figure 3:**
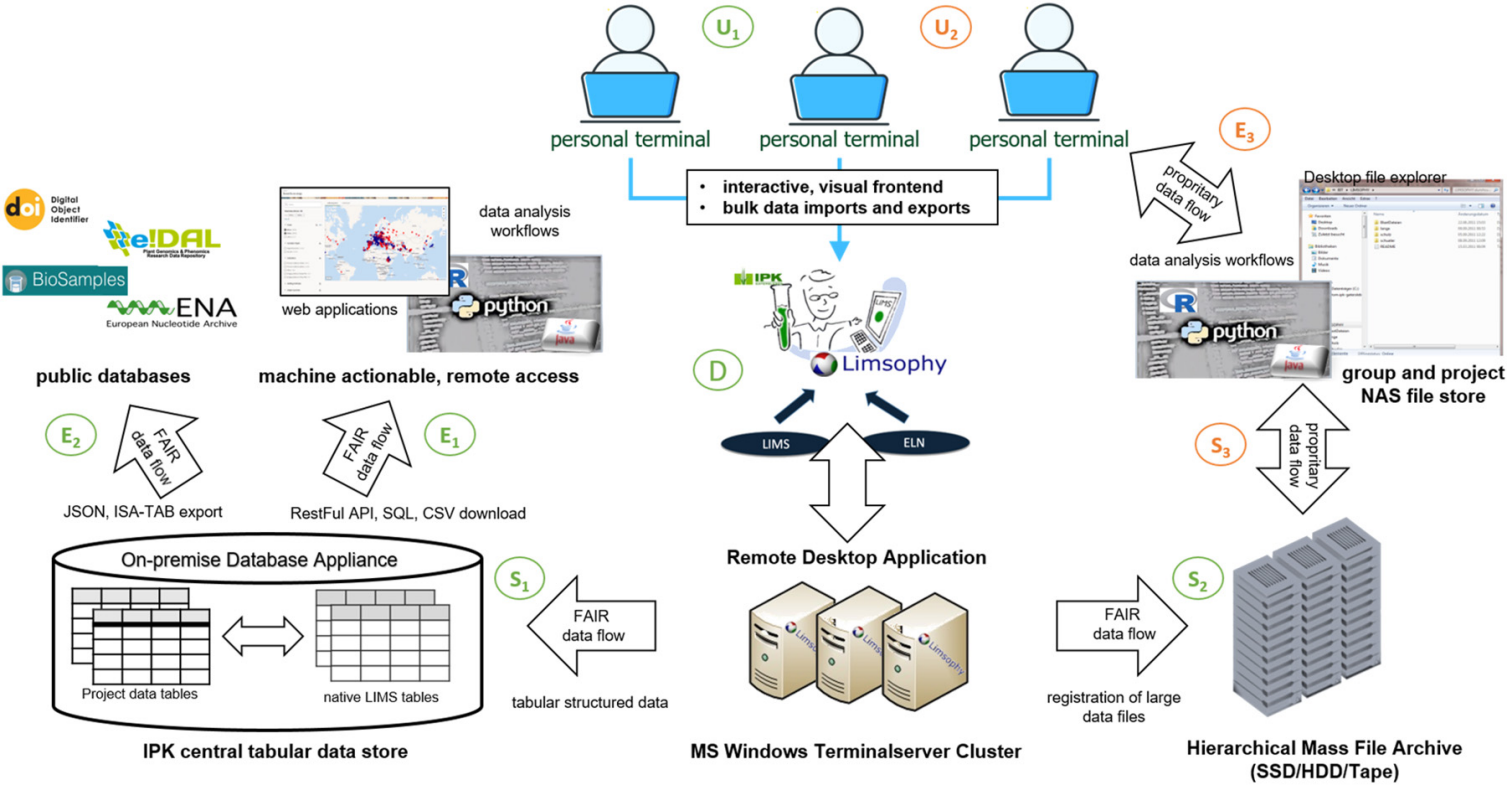
System integration architecture of the IPK RALIMS in 2024: The user front-end component (U), the RALIMS data management software (D), the storage infrastructures (S) and the data export and data access interfaces (E) are divided into a data flow following the FAIR principles (highlighted in green) and components adapted to the needs of proprietary data flows, such as sensitive data (highlighted in orange). The lower indices indicate the instance of the respective system component that features specific functionalities, which are more closely indicated by the data flow arrow.

In completion of the ISA core, the entity-attribute-value (EAV) model is applied, which is a venerable method for representing arbitrary information on an object. According to the current stored data, the ISA core covers about 80 % of the use cases and can be implemented efficiently in well storage and access optimized RDBMS backends. Specifically, the IPK ORACLE RDBMs backend features a robust relational storage engine in large-scale environments. As industry standard, it features in-build performance optimisation technology such as partitioning, bitmap index, query vectorisation, in-memory structures query, caches etc. To combine this relational model based-based structures with no SQL elements, attribute value extensions, large binary objects, data streams, graph data structures, external files or JSON and XML document data types are supported as well. The support of hybrid data structures is the core pillar and its well optimised implementation in ORACLE database stack enables to host data of any use case and ensure scalability and efficient operation over millions of data points [6]. Figure 3 shows the current, system-integrated architecture of the RALIMS research data infrastructure a decade after its initial deployment.

Over the past decade, IPK software engineers have developed complementary components such as BrAPI [7], a RESTful remote application programming interfaces and exposed SQL based interface to query tabular data [5], database stored procedures to connect to the DataCite API [8] to mint DOIs as permanent unique and globally resolvable data set identifier, and options for exporting FDO-compliant datasets, such as an ISA-TAB and their publication, for example in EMBL BioSamples [9] and ENA [9] or e!DAL-PGP [10]. In addition, structures for referencing the controlled vocabulary in cross domain ontologies [11], such as the NCBI taxonomy and plant ontology, and for mapping to plant specific metadata standards, such as MIAPPE [12], were implemented. Finally, a system integration with IPK genebank information system [13] was implemented to ensure harmonised material and sample management.

## 3 Dovetailing with data management for service and research processes

**Figure 4:**
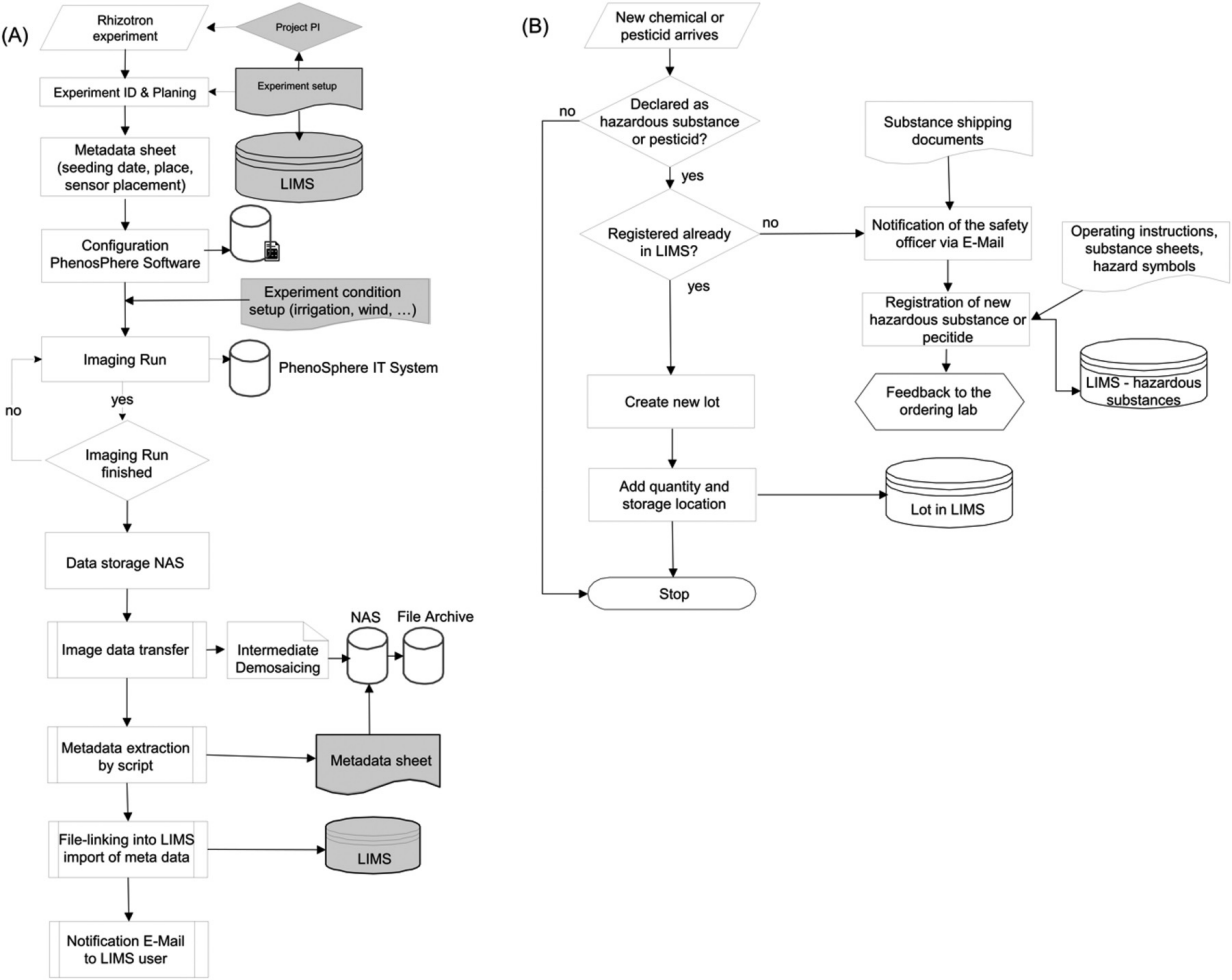
UML activity diagram of IPK rhizotron phenotyping (A) and chemicals management (B).

The aforementioned system architecture serves two major classes of data management processes of the IPK. The first category are sole **service processes** for centrally managed instruments that are utilized in research projects. They follow an institutional agreed process for primary data capture and are operated in an order-processing manner by IPK financed permanent staff. Examples are data acquisition processes like the high-throughput sequencing and phenotyping processes [14] or unpublished internal service processes like root phenotyping in the rhizotron system of IPK’s whether simulation facility ‘PhenosSphere’ and chemical management as shown in Figure 4.

Both comprise (a) defined personnel and organizational responsibilities including defined transition points between the laboratories, the scientist and the LIMS project team as well as (b) defined standard-compliant and machine-processable data formats, (c) mandatory metadata standards, and (d) previous defined data publication process for sequence data and for phenotyping data.

An exemplar phenotyping process, implemented as a service process in LIMS is the scoring of plant traits in green houses or on fields. Here data capture using the smartphone app PhenoApp [14] is the start of a LIMS data flow. The clearly designed and easy-to-use app could be integrated well into the data capture process. LIMS enables users to create input files and methods for the evaluation. Methods that have already been described can be selected again and/or reused in a modified form. Different genotypes so called accessions from the oil and fodder plant assortments are assessed in various trials. The research data is recorded exclusively using the app. This includes continuous recording with scoring values or the linking of images with scoring values. Another advantage is the ability to take photos directly with the app for documentation purposes.

Another area of application for the IPK-LIMS concerns the documentation of all work with genetically modified organisms (GMOs). Documentation of GMOs is essential to achieve scientific goals and to promote safety, transparency and trust in the responsible use of biotechnology. In general, work with GMOs is subject to strict control, regulated by corresponding laws and controlled by state administrative offices. To ensure the safety and documentation of GMO work at IPK, the LIMS has a GMO module which can document all GMO-relevant data, from generation, storage (room lists), work carried out (cultivation, harvest) to the destruction of the corresponding GMOs. Data access is personalised and protected, and entries and changes are traceable. Each project leader has full access and data entry rights for his or her own (laboratory) area, but not for other working groups. There are detailed lists with all relevant information such as the type of GMO and its safety level (S1 or S2 according to the Genetic Engineering Safety Ordinance), selection markers, donor and recipient organism (species), storage location, purpose of use in specific scientific projects and project leader. Information about specific GMOs can be exchanged between working groups. This step is a prerequisite for another working group to gain access to the corresponding GMO. The LIMS also allows the automated creation of documentation (‘annual reports’) in accordance with the German Genetic Engineering Recording Act. This type of documentation at IPK has been fully evaluated and approved by the responsible State Administration Office in Halle/Saale. The GMO module in LIMS also allows the organised storage of documents, letters, room plans, correspondence, etc. that characterise the respective project area. A repository of this kind would not be feasible without the security features provided by a LIMS. It therefore serves as a benchmark for other institutions that work with GMOs. In summary, the IPK LIMS (1) meets legal and regulatory requirements, (2) ensures traceability and control, and (3) guarantees the IPK’s liability and responsibility towards the environment and society.

In contrast, data flows in **research projects** need to be more agile and are less rigidly structured, reflecting the nature of innovation-driven science. Here, the mentioned core service processes are dovetailed with the immersive analytics driven knowledge generation in research projects [18]. An example is BRIDGE [15] a research project for the genotypic and phenotypic characterisation of barley samples from German Federal Ex situ Genebank of plant genetic resources [13], a research project for the genotypic and phenotypic characterisation of more than 22 thousand barley accessions of the IPK genebank.

Here, the pre-defined RALIMS service processes sequencing, seed management and scoring process were applied and interweaved to manage more than 48,000 samples from sequencing and cultivation with about 776,000 data points.

Such interweaving of sole services processes and project specific ones is a joint activity of project and core service staff with a high demand of a very close interaction. The data are exposed via SQL views to the RALIMS data backend through a web portal [16]. These and other projects, with a total of more than six million samples and terabytes of data, are incubators for building the capacity to provide FAIR RDM processes to networks such as the European life-sciences infrastructure for biological information (ELIXIR) [17] or at national level in the German Bioinformatics Network (de.NBI) [18] or the National Research Data Infrastructure (NFDI) (https://www.nfdi.de) in the consortia, FAIRAgro [19] and NFDI4Biodiversity [20].

The third category are **hybrid service processes**. Those share common steps and data structures, but are more agile and driven by individual and project set-ups. Examples are the integration of Electronic Lab Notebook (ELN) documentation or archival of imaging, like microscopy. Here we have shared process elements, like documentation of experimental set-ups, measure methods, documentation of material and sample preparation. The documentation and sharing of experimental results and used processing and data analysis pipelines need to be supported in a flexible less strict way.

Prominent example at IPK are microscopy and the complex metabolomics lab work flows. For example, different microscopes produce varying types and amounts of images, with Lightsheet Fluorescence Microscopy being a notable case. This technique is ideal for long-term live-cell imaging and/or imaging of large samples, often generating relatively few but extremely large files, some exceeding one terabyte. Such structured data capture processes across several dozens of instruments [21] requires a well-designed research data flow into backend storage and the documentation of the measured object and images taken. In order to ensure FAIR storage and handling, the following steps are implemented. Image nomenclature follows a naming convention consisting of an image number, followed by date and time automatically stamped during acquisition, representing the first unambiguous identifier. Given the large data size, initially images are stored locally during experimental procedures. Once decided that the images are of good quality, they are transferred to a filer from where images will be transferred into HSM, respecting a user-defined folder hierarchy. At the same time, metadata associated with each image is recorded by manually adding entries into a dedicated module within LIMS created specifically for this microscope. Which metadata is recorded was defined after four weeks of microscope use, and include information about the user, e.g. name, cost centre, sample, e.g. species, organ, transgenic unique GMO number in the LIMS GMO documentation module, image-specific metadata, e.g. type of experiment, fluorescence colours detected and associated proteins or stains, and the file name and the file path in HSM. Upon entry creation, LIMS creates an unique identifier that will be associated with the image. Only raw data is stored, given the size of the files, and that processed data can be easily regenerated. By referring to the acquisition date in the LIMS entry and/or in the image name, it is straightforward to refer to the corresponding entry into the ELN. There, extended information on the experimental setup, and on image processing steps are documented. All in all, this integrated approach leverages LIMS as a central hub, ensuring microscopic data is managed in a FAIR manner by combining modules for GMO, ELN, and imaging-specific data.

Another example is the documentation of metabolomics data in an electronic laboratory notebook (ELN). In context of such more semi-structured documentation, it is essential to follow best practices to ensure data integrity, reproducibility and compliance with FAIR principles. The ELN must first have a user permissions hierarchy to protect sensitive data. Standardized metadata fields and naming conventions are essential to maintain consistency and facilitate data retrieval [22]. Users create templates based on experimental entries that are tailored to the type of methods used (i.e. GC-TOF MS data vs UPLC-TOF MS or UPLC-DAD/FLD). These templates include experimental design parameters, their procedures, reagents used, as well as sample preparation, equipment utilised, special observations and intended downstream analysis procedures and statistical tests. It is imperative to separate the raw data from those data that are run through any analysis pipelines. All data entries are time stamped and attributed to those responsible for the running of the instruments and analysis of the data in order to maintain a clear audit trail. In our experience, leveraging an ELN that is accessible institute-wide enhances the collaboration and data sharing between and within individual groups. The metadata augmented files can then also be used for downstream reporting in standard formats [23] and submission to the proper metabolomic repository databases, like GNPS, or MetaboLights [24].

## 4 Lessons learned from a decade of centrally organised research data management infrastructure

**Figure 5:**
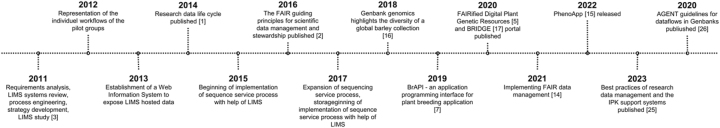
History of activities for a harmonised research data management using RALIMS at the Leibniz institute of plant genetics and crop plant research.

The establishment of a centralised technical infrastructure for research data management and digitally valid documentation of scientific experiments with the installation of the RALIMS technology platform in 2011 was the beginning of a process for FAIR data management at the IPK that continues to this day. Figure 5 show the actions and refinements over a decade to align to the requirements of the multidisciplinary research landscape at IPK in alignment with the international RDM ecosystem.

These activities can be subdivided into three categories: (a) actions to embed the system in the laboratory and research processes, (b) the continuous refinement and supply of technical features and (c) training programmes. Subsequently, an excerpt is given of the major lessons learnt in more than a decade of LIMS-based research data management and its effect for IPK’s sustainable but agile research data management infrastructure are discussed.

Centralisation of RDM is linked to the need for a **strong cross-department and group communication**, e.g. to establish best practices, standard operating procedures, and build confidence in the benefits of centralisation. In this context it became apparent how important it is to do this in a participatory process in a collaborative development. The basis for the establishment of a central RDM infrastructure across domains and organisational structures are well-chosen pilot working groups as seedlings for the step-by-step roll-out in order to achieve the highest possible level of acceptance among the majority of employees and overcome a certain scepticism and fear of complex learning processes. Specifically, it was beneficial to emphasize the added value for daily work and to promote trusting communication at eye level through joint workshops and trust-building on a personal level with a high degree of social competence in order to discuss issues across all hierarchies. One example of how a technical solution that could be implemented at an ad hoc basis made daily routine work considerably easier was the launch of a centralised inventory for chemicals and hazardous substances in RALIMS. Thanks to this integrated catalogue across all laboratories, previous emails to all enquiries were no longer necessary.

The next lesson was the importance of **customizability and configurability** of the RDM infrastructure at user level without having to manipulate the software code. Individual views to the same underlying RDM infrastructure must be supported. This ranges from a customisable front-end layout to individual data import interfaces that meet the needs of the data curators. For example, we have licensed a RALIMS module that allows us to design customised GUI modules and macros to replace repetitive sequences of GUI interactions, simplifying workflows and making them more compact. By August 2024, over 70 of custom modules have been designed, resulting in higher user acceptance compared to the out-of-the box front end. We have also used the ability to design modules to create an electronic laboratory notebook (ELN) that can be used by all users equally, but can also be customized to the needs of the individual practice.

**Figure 6:**
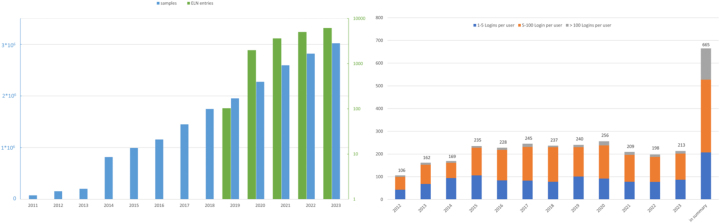
The left-hand figure illustrates the steady growth of the data recorded by the annual number of new sample and lab book entries since 2011. For reason of a visual clarity, further data points, such as the data assigned to samples or referenced files, are not shown. The absolute number of these data points is of course substantially higher than the number of samples. For example, by July 2024, 1,067,738 results and 2,375,928 files are linked to samples in the LIMS. On the right-hand side, the number of different users is shown for comparison, broken down by activity class per year (<5 logins, 5–100 logins, >100 logins per year).

Finally, yet importantly, are **agile training concepts** and formats in addition to classic classroom training, like hands-on training and open user meetings. Here, the interaction among trainees from the various research groups were stimulated. Moderated by the LIMS managing team, senior LIMS users give impulse presentations on selected topics, LIMS function modules, best practice tips and examples of data acquisition processes in the labs. A further format to push users to share their individual experiences and practices, as well as interest to provide LIMS open hours as regular consultation sessions, on a monthly basis. Finally, special training formats were suggested and implemented, like multilingual tutorial videos ranging from general system operation to service processes, multi-day summer schools [25]. All of these measures have contributed to the growing acceptance as shown in Figure 6.

## 5 Outlook – roadmap data management

Operational and stewardship issues are topics that will continue to be addressed in the coming years. These include standardized materials management and a digital twin concept at local or international scope [26]. Technical measures relate for example to feature the export of FAIR Digital Objects (FDO) as actionable knowledge units [27], such as RO-Crate [28] and ARCs [29]. Technical improvements will be done to support distributed object store technology [30] as storage backend to make the very extensive raw data more efficiently accessible and to support its use in cloud environments.

The RDM processes implemented in RALIMS will be further enhanced to meet recent recommendations of a RISE-DE [31] self-evaluation, such as an integration with project management, research data management plans, an interlink to publication processes managed in IPK library. Another focus in the area of user training is the integration into national and international training programmes, for example in de.NBI, NFDI or ELIXIR. Finally, the integration of instruments, the import of complex data and the creation of cross-workgroup templates and intuitive input masks are essential, including the use of modern GUI design and further support for mobile devices.

All the activities described above require resources, and it is always a balancing process between the excellence of the RDM, the breadth and depth of the experiments, the intensity of the analyses, and the time needed to interpret the results. Optimizing this balance is an ongoing task with the aim of continuously improving the quality of research. The decision to use a commercial solution as the central infrastructure was driven by the experience gained in the five-year funded German bioinformatics cluster ‘BIC-GH’ (2002–2007), supplemented by a literature study and by comparison with institutions with a comparable profile, staff composition and size, number and complexity of RDM processes and service quality obligations such as infrastructure availability and personnel’s RDM literacy. A detailed retrospective cost calculation could be useful as follow-up work to develop a detailed cost-benefit analysis to evaluate the decision made at IPK. This could compare the two hypothetical models that were not implemented with the IPK and the model that was actually chosen. One approach to estimating the costs of the hypothetical operating models could be to analyse facilities that have opted to implement these models.
